# Trait‐Based Drivers of Host Specificity in Freshwater Mussel–Fish Parasitic Interactions

**DOI:** 10.1002/ece3.74097

**Published:** 2026-07-28

**Authors:** Irene Sánchez González, Andrew W. Park, Krista A. Capps

**Affiliations:** ^1^ Odum School of Ecology University of Georgia Athens Georgia USA; ^2^ Savannah River Ecology Laboratory University of Georgia Aiken South Carolina USA

## Abstract

Species interactions, particularly parasitism, are fundamental drivers of biodiversity and ecosystem function. Life history strategies, defined by differences in reproductive output, growth, and lifespan, play a key role in shaping host–parasite relationships. Freshwater mussels (Family: Unionidae) are a diverse and highly imperiled group that rely on an obligate ectoparasitic larval stage on host fishes. Despite this dependence, they are often excluded from general parasitology frameworks and differ from typical parasites due to their long and variable lifespans. In this study, we integrated publicly available data on mussel and fish phylogenies, life history traits, and host–parasite associations to evaluate patterns of host phylogenetic specificity in North American mussels. We tested whether mussel life history strategies predict differences in host phylogenetic breadth. Our results reveal that host phylogenetic specificity is phylogenetically conserved but variable among mussel species. Mussel life history traits were related to host use: equilibrium strategists (long‐lived, low fecundity) disproportionately parasitize short‐lived, opportunistic fishes; opportunistic mussels (short‐lived, highly fecund) favor longer‐lived, equilibrium hosts. Periodic strategists show intermediate traits and more variable patterns. These patterns suggest underlying ecological and evolutionary trade‐offs in parasitic strategies, offering new insights into host–parasite dynamics and providing valuable information for conserving mussel–fish interactions and ecosystem function.

## Graphical abstract

Freshwater mussels (Unionidae) rely on an obligate parasitic larval stage on fish hosts, yet their host specificity remains poorly understood in a broader ecological and evolutionary context. By integrating phylogenetic, life‐history, and host‐use data, we show that host phylogenetic specificity is conserved but varies systematically with mussel life‐history strategies and reproductive traits. Our results reveal non‐random associations between mussel and fish life histories, highlighting trait‐based trade‐offs that shape host–parasite interactions and offering new insight into the ecology and evolution of parasitism.

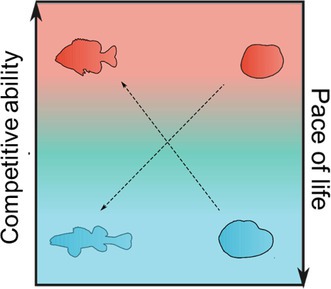

## 1 Introduction

Species interactions are fundamental drivers of biodiversity and ecosystem function (Schwartz et al. 2000; Balvanera et al. 2006; Slade et al. 2017). Antagonistic, mutualistic, and commensal relationships shape patterns of species distribution, abundance, and ecosystem stability by influencing survival and reproduction (Mougi and Kondoh 2012; Qian and Akçay 2020). These interactions underpin community structure and dynamics, with far‐reaching implications for biodiversity conservation and ecosystem resilience (Heinen et al. 2020; Valiente‐Banuet et al. 2015).

Parasitism is one of the most common and influential ecological interactions (Price 1977; Lafferty et al. 2006). Parasite–host relationships are often highly specialized and complex, shaped by adaptations that allow parasites to exploit hosts while bypassing their defenses (Ewald 1995; Turner et al. 2021). These interactions regulate host population sizes, alter behavior, and drive evolutionary change (Buckingham and Ashby 2022; Papkou et al. 2016; Watson 2009; Hughes and Libersat 2019). In turn, hosts can evolve a range of mechanisms to tolerate infection, balancing the costs of parasitism with survival and reproduction (Boots et al. 2008; Budischak et al. 2018). Beyond individual hosts, parasitic relationships can have cascading effects on entire ecosystems by influencing food web dynamics, nutrient cycling, and community composition (Frainer et al. 2018; Hatcher et al. 2006).

Life‐history traits of both hosts and parasites are central to shaping parasitic interactions (Koella et al. 1998). Life‐history theory describes how organisms allocate limited resources among growth, reproduction, and survival over their lifespan (Capdevila et al. 2020; Ferreira et al. 2023; Salguero‐Gómez et al. 2016; Verberk et al. 2008). Across taxa, longer‐lived species tend to be stronger competitors. In stable conditions, they are more efficient at acquiring and using resources and often specialize in the most profitable or high‐quality ones. In contrast, shorter‐lived species tend to adopt generalist strategies to maximize resource use (Grime 1977; Winemiller and Rose 1992; Dennis et al. 2011). Host life‐history strategies influence susceptibility to infection: fast‐paced hosts, with short lifespans and high reproductive rates, often invest less in immune defenses and are more vulnerable to parasites. In contrast, slow‐paced hosts, with longer lifespans and lower reproductive rates, typically allocate more resources to resistance or tolerance mechanisms (Sears et al. 2015; Agnew and Koella 1999; Johnson et al. 2012). Parasites exhibit analogous trade‐offs: species with “fast” life histories, characterized by high fecundity and rapid reproduction, tend to exploit a broader range of hosts, whereas parasites with “slow” life histories, investing more in prolonged development or specialized infection strategies, often exhibit narrower host ranges (Manzoli et al. 2021; Park et al. 2018; Mysterud et al. 2015). Parallel dynamics in host and parasite life histories are key to understanding coevolution, host specificity, and infection success.

Host specificity, defined as the degree to which a parasite relies on particular host species, has broad ecological and evolutionary consequences (Poulin 2006; Kirchner and Roy 2000). Parasites vary along a continuum from specialists, which infect one or a few hosts, to generalists, which exploit a wide range of hosts (Šimková et al. 2006; Tripet and Richner 1997; Hellgren et al. 2009). Generalist parasites benefit from broad host availability, increasing opportunities for transmission, but often face trade‐offs such as suboptimal exploitation, maladaptive virulence, or reduced transmission efficiency in any single host. In contrast, specialists may achieve higher infection success and transmission within their preferred host but are more vulnerable to environmental disruptions that affect host availability (Johnson et al. 2019; Sasal et al. 1999; Manzoli et al. 2021). Host specificity is conventionally quantified by the taxonomic or phylogenetic breadth of host species a parasite can infect, with phylogenetic analyses offering a powerful framework to assess patterns beyond raw host counts and to predict potential host–parasite associations among other species (Poulin and Mouillot 2005; Park et al. 2018).

**FIGURE 1:**
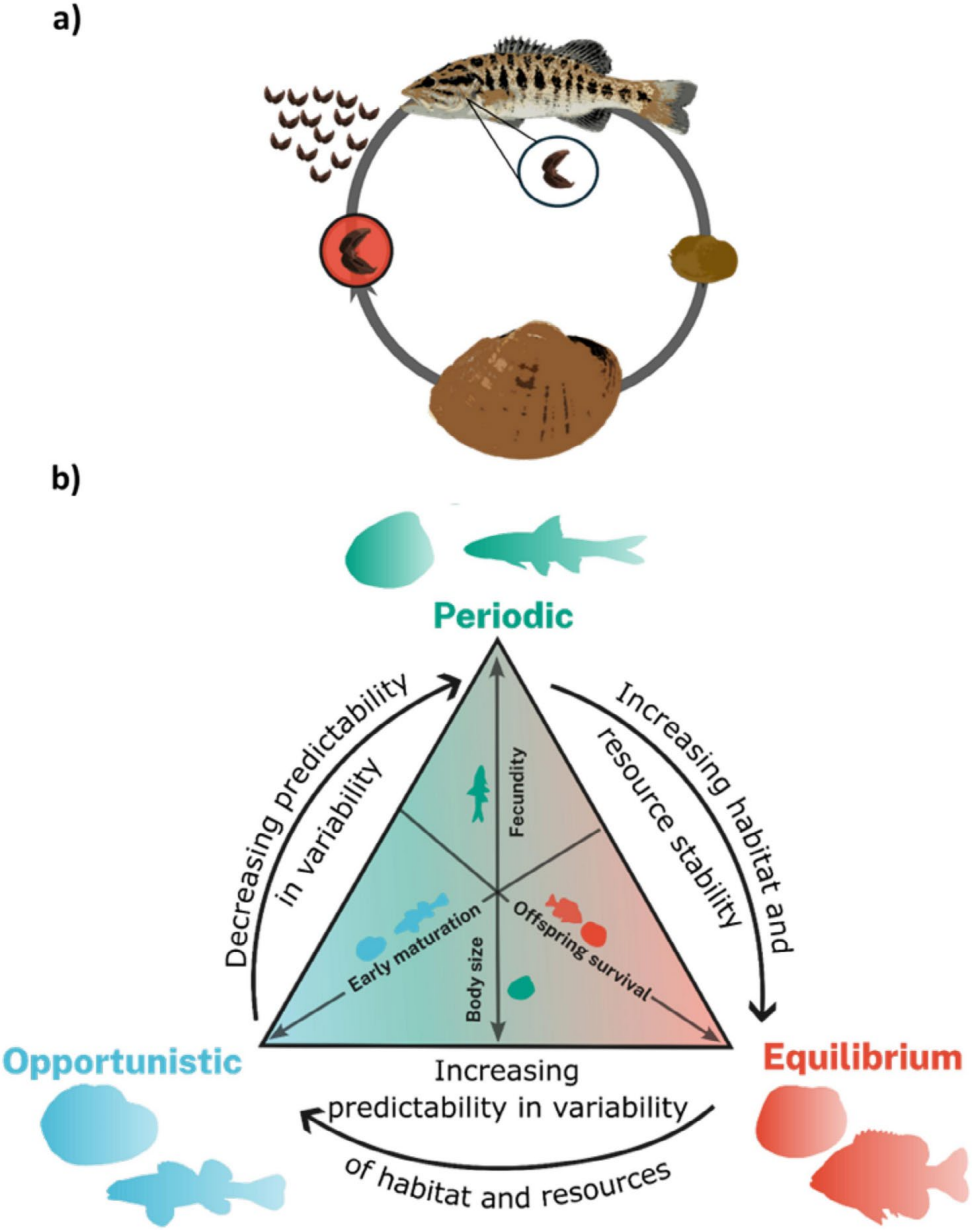
(a) Freshwater mussel life cycle. The parasitic larval stage (glochidia) is released by female adult mussels and must attach to a suitable fish host (top center) for development. After metamorphosis, juvenile mussels detach and settle into the substrate. (b) Triangular life history framework for freshwater mussels and fish. Exterior curve arrows indicate optimal habitat for each life history strategy. Interior, straight arrows indicate characteristic traits for each life history strategy. Interior silhouettes symbolize if a trait is applicable to mussels, fishes or both. For all arrows, the direction in which the head(s) of the arrow(s) is pointing indicates a positive relationship with the term(s) associated with the arrow. For example, periodic fishes and mussels are characterized by living in habitats with great environmental disturbance and limited stability. Periodic fishes are characterized by high fecundity and periodic mussels are relatively large‐bodied.

Freshwater mussels (Order: Unionida) represent a unique and ecologically significant example of parasitism among invertebrates. Although largely sedentary as adults, their life cycle critically depends on a parasitic larval stage on host fishes for dispersal upstream (Barnhart et al. 2008; Denic et al. 2015; Terui and Miyazaki 2015; Figure 1a). Their life cycle begins when males release sperm into the water, which females filter to fertilize eggs internally. These eggs are brooded in specialized gill pouches and later released as parasitic larvae called *glochidia* (Haag 2012). To survive, glochidia must attach to the gills, fins, or skin of freshwater fishes, or occasionally amphibians, where they encyst, transform into juveniles, and eventually drop off to the substrate (Rogers‐Lowery and Dimock 2006). Failure to attach results in larval death, highlighting the critical role of host encounter. To facilitate host infection, mussels have evolved diverse strategies, including mantle lures that mimic prey, conglutinates that package glochidia into bait‐like structures, and simple broadcasting of larvae into the water (Barnhart et al. 2008). This obligate parasitic phase on a single host species during their larval development classifies mussels as partially monoxenous ectoparasites (Rock et al. 2022). Globally, there are 976 recognized freshwater mussel species (Graf and Cummings 2021), but documented host interactions exist for only 185 mussel species and 386 fish host species, with a strong bias toward North American systems (Hopper et al. 2025).

Despite their parasitic larval stage, freshwater mussels are often overlooked in general parasite ecology frameworks and databases (Hoffman 2023). They also stand apart from most parasites due to their extended and diverse lifespans, ranging from five to 100 years (Haag 2012). Nearly 70% of North American mussel species are now imperiled (Haag and Williams 2014), making them a rare case of a parasitic organism at the center of conservation concern. Their dependency on host fishes for development and dispersal makes them vulnerable to declines in host populations and habitat fragmentation (Schwalb et al. 2013; Vaughn and Taylor 2000). Beyond their parasitic stage, mussels function as ecosystem engineers, filtering water and creating biogeochemical hotspots (Atkinson and Vaughn 2015; Atkinson et al. 2017; Vaughn 2018) and creating habitat for other aquatic organisms (DuBose et al. 2024; Hopper et al. 2019; Sansom, Atkinson, and Bennett 2018). As such, unionid mussels represent an underexplored but diverse system linking parasitology, community ecology, and conservation biology.

Fishes and mussels exhibit contrasting life history strategies. Fishes are typically shorter‐lived (2–5 years) and highly mobile (Hedden and Gido 2020), while mussels are sedentary filter feeders with longer lifespans (up to 100 years) (Haag 2012; Sansom, Bennett, et al. 2018). Despite these differences, both groups can be categorized using the trilateral equilibrium–periodic–opportunistic (E–P–O) life history framework, which captures distinct strategies along a continuum of lifespan, age at maturity, and reproductive output. Opportunistic species (O) resemble “r‐selected” species and exhibit a faster pace of life, with short lifespans, early maturation, and high fecundity, thriving in highly variable habitats. Equilibrium species (E) share traits with “K‐selected” species and have a slower pace of life, including long lifespans, delayed reproduction, and low fecundity, favoring stable, competitive environments. Periodic species (P) occupy an intermediate position, combining moderate lifespan, delayed maturation, and intermediate fecundity, and are often adapted to seasonal or predictably fluctuating habitats (Haag 2012; Winemiller and Rose 1992; Figure 1b). For fishes, these categories are mainly defined by their age at maturity, level of parental care, and fecundity (Frimpong and Angermeier 2009; Winemiller and Rose 1992). Opportunistic species mature early with little parental care. Periodic species grow fast, produce many offspring, show minimal parental care, and mature later. Equilibrium species mature late, investing more in parental care leading to higher juvenile survival, but having fewer offspring. Mussels, on the other hand, show a slightly different expression of these strategies (Haag 2012). Opportunistic mussels grow quickly, have shorter lives, reach a moderate to large size, and produce many offspring. Periodic mussels have moderate lifespans, mature younger, are smaller, and have lower fecundity compared to opportunists. Equilibrium mussels live a long time, mature slowly, and have a moderate to large size but produce fewer offspring.

Integrating life‐history strategies of fishes and mussels can improve predictions of host–parasite interactions and their underlying mechanisms. Here, we assess host specificity across North American freshwater mussels by integrating host‐use data with life‐history strategies and phylogenetic information for both mussels and their fish hosts. We test whether life‐history strategies predict differences in host breadth and whether these patterns reflect trait‐based trade‐offs in parasitic strategy, while incorporating host phylogeny to refine estimates of specialization beyond raw host counts.

We hypothesize that host specificity in freshwater mussels is influenced by life‐history strategy and reproductive traits, reflecting trade‐offs in resource allocation and transmission. We predict that opportunistic mussels exhibit broader host use (lower phylogenetic specificity), equilibrium species are more specialized, and periodic species are intermediate. We further predict that transmission traits mediate these patterns: highly fecund species and those using broadcast infection have lower host specificity than those using targeted strategies (e.g., mantle lures or conglutinates).

**FIGURE 2:**
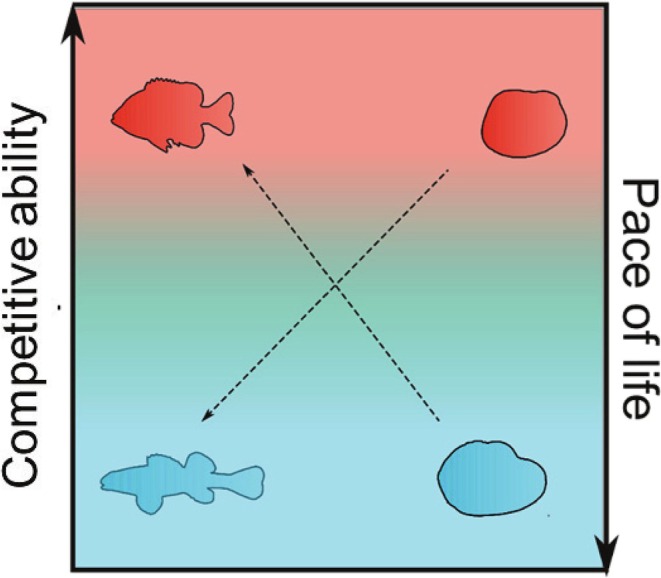
Conceptual model illustrating trait matching between mussel and host fish life history strategies. Red represents equilibrium strategists, green periodic and blue opportunistic. Life history strategies are positioned along a trade‐off axis from highly competitive ability and slow pace of life (low fecundity, slow growth) in the upper left (orange) to low competitive ability and fast pace of life (high fecundity, rapid growth) in the lower right (blue). Dashed arrows indicate expected patterns of trait matching, where equilibrium mussels are more likely to use opportunistic fish hosts, and opportunistic mussels tend to use equilibrium fish hosts.

We also hypothesize that mussel and fish life histories are non‐randomly associated along shared gradients of competitive ability and pace of life (Figure 2). Equilibrium mussels (slow, competitive) are predicted to disproportionately use opportunistic fish hosts (fast, low competitive ability), whose high turnover and influx of immunologically naïve individuals increase susceptibility and provide a consistent pool of infectable hosts. In contrast, opportunistic mussels (fast, highly fecund) are predicted to more frequently exploit equilibrium fish hosts, which are longer‐lived and demographically stable, offering more predictable conditions for larval development despite stronger immune defenses. Periodic mussels are predicted to show intermediate or mixed associations. These predictions suggest mussel–fish interactions deviate from random expectations and reflect trade‐offs between host susceptibility and parasite transmission strategy.

## 2 Methods

### 2.1 Fish Host‐Mussel Parasite Data Compilation

We compiled a list of freshwater mussel species with known fish hosts using the SHEL‐D trait database (Hopper et al. 2023). This integrates literature from the Freshwater Mussel Host Database (https://mollusk.inhs.illinois.edu/57‐2/) documenting 1170 unique associations between the glochidia of 167 North American mussel species and 247 host fish species through both laboratory and natural observations. The dataset only reports unique mussel–fish species pairs, excluding duplicate records of the same interaction reported across multiple studies in the original Freshwater Mussel Host Database. The Freshwater Mussel Host database is a widely recognized and frequently cited resource among researchers and conservation practitioners working in freshwater mussel ecology. Host associations are categorized based on the type and outcome of the interaction: lab infestations (LI), where attachment occurs under experimental conditions but metamorphosis is not observed; lab transformations (LT), where metamorphosis to the juvenile stage occurs in the lab, although the distinction between primary and marginal hosts is not made; natural infestations (NI), where wild‐caught fishes are found with attached glochidia but without confirmed metamorphosis; natural transformations (NT), where successful metamorphosis is observed in natural settings or inferred from advanced encapsulation; zero transformations (ZT), where attachment fails to result in development; and cases with no specified outcome (NS). For this study, we focused exclusively on lab transformations, natural infestations, and natural transformations, as these categories provide the strongest evidence of functional compatibility between mussels and fish hosts. We excluded lab infestations, as the absence of metamorphosis under controlled conditions offers limited support for host suitability in the wild.

### 2.2 Fish Phylogeny and Calculation of Host Phylogenetic Specificity

We quantified host phylogenetic specificity for each mussel species using a published fish phylogeny from the Fish Tree of Life (Chang et al. 2019), following the methods described in Park et al. (2018). Analyses were conducted in R 4.3.2 (R Core Team 2023) and Rstudio 2024.09.0 (Posit Team 2024) using the R package *ape* (Paradis et al. 2004). Mussel–fish association data were compiled into a binary matrix, where columns represented fish hosts and rows represented mussel species. Fish species names in the association matrix were standardized to match the phylogeny, resolving taxonomic discrepancies using synonyms obtained via the R package *rfishbase* (Boettiger et al. 2012). Pairwise phylogenetic distances between fish species were computed from the phylogenetic tree using cophenetic.phylo in the *ape* package. We then calculated the standardized effect size (SES) of the mean pairwise phylogenetic distance (MPD) among host fish for each mussel species using the ses.mpd function in the R package *picante* (Kembel et al. 2010). Randomizations (*n* = 1000) were performed using the independent swap algorithm (SIM 9, Gotelli 2000), which maintains the marginal totals of the community matrix. We selected *SIM 9* as the primary null model because it simultaneously controls for variation in mussel host breadth and differences in host sampling intensity, ensuring that observed patterns of phylogenetic clustering reflect biological structure rather than sampling bias. Standardized MPD z‐scores indicate whether mussel host sets are more (negative values) or less (positive values) phylogenetically clustered than expected by chance. Mussels associated with fewer than two host species were excluded from this analysis due to insufficient data to estimate MPD.

To assess robustness, we compared SIM9 to three null models with different ecological constraints. *SIM1* (equiprobable; sample. pool in picante) assumes all mussels and fish hosts are equally likely. *SIM2* (row sums fixed; frequency) preserves the number of hosts per mussel species, controlling for host breadth while treating all fish as equally suitable. *SIM3* (column sums fixed; richness) preserves the number of mussels per host fish while treating all mussels as equally likely to use any host. Comparing SES MPD across SIM1, SIM2, SIM3, and SIM9 provides a sensitivity analysis showing that observed phylogenetic clustering of mussel hosts is robust to null model choice.

### 2.3 Life History Classifications of Fish and Mussels

We classified fish and mussel species into three life history (opportunistic, periodic, equilibrium) strategies based on previous literature (Haag 2012; Mims and Olden 2013; Moore et al. 2021; Perkin et al. 2017; Tables S1 and S2) or, for species lacking trait data in the literature, we inferred life history strategy based on phylogenetic relatedness, assigning species to the same strategy as closely related congeners. Quantitative trait data or previous literature classification were available for 30% of mussel species and 45% of fish species; these served as the basis for phylogenetically informed assignment of life‐history strategies (but not quantitative trait values) for the remaining species. Life‐history traits are often phylogenetically conserved, with closely related species exhibiting similar combinations of traits (Winemiller 2005; Haag 2012). Phylogenetic inference has been successfully applied in comparative studies in other taxa to address missing data while preserving underlying ecological patterns (Soria et al. 2021; Penone et al. 2014).

### 2.4 Phylogenetic Signal of Traits and Life‐History Strategies

We evaluated whether key mussel traits used in life history classifications (fecundity, age at maturity, and maximum lifespan) and host phylogenetic specificity exhibited phylogenetic signal using Pagel's λ test. Trait data were log‐transformed and matched to a published phylogeny of freshwater mussels from Keogh et al. (2024). We used the original topology and pruned it to include only species in our trait dataset. Because species coverage varied among traits, the number of taxa differed across analyses: 98 species for host specificity, 68 for fecundity, 84 for maximum age, and 45 for age at maturity. All phylogenetic analyses used the corresponding pruned trees. Using the R packages phylobase, adephylo (Dray and Jombart 2009), and phytools (Revell 2024), we computed Pagel's λ to measure the extent to which trait variation is explained by shared evolutionary history, with values near 1 indicating strong phylogenetic dependence and values near 0 indicating phylogenetic independence. This approach enabled us to assess the degree to which life‐history traits are phylogenetically conserved, while also examining patterns of host specificity across the mussel phylogeny. We visualized the phylogeny including only the species in our study and mapped both fecundity and host specificity onto the tree to explore trait variation in a phylogenetic context (Figure S1).

### 2.5 Mussel Life History Strategy and Host Specificity

To evaluate host specificity and life‐history patterns in freshwater mussels, we conducted both standard and phylogenetic analyses. Because phylogenetic data were incomplete, these analyses used smaller subsets of species but accounted for shared evolutionary history. In contrast, standard analyses maximized sample size and statistical power. To evaluate differences in host specificity across mussel life history strategies, we first tested for normality of the residuals using the Shapiro–Wilk test (R function: *Shapiro.test*) and for homogeneity of variances using Levene's test (R function: *leveneTest*). As data met parametric assumptions (Shapiro–Wilk: *W* = 0.968, *p* = 0.230; Levene's test: *F* = 1.50, *p* = 0.234), we conducted a one‐way analysis of variance (ANOVA) to test for differences among life history groups. We had life history and specificity data for 48 mussel species. To assess this relationship while accounting for shared evolutionary history, we also conducted a phylogenetic ANOVA using the function *phylanova* in the package *phytools* (Revell 2024). This analysis was restricted to 41 species for which overlapping data on life history, host specificity, and phylogeny were available. To examine whether specific life history traits are related to host specificity, we selected one categorical trait (host infection strategy) and one quantitative trait (fecundity) from the SHEL‐D freshwater mussel database (Hopper et al. 2023). Among the freshwater mussel traits available, fecundity and host infection are the most directly linked to host–parasite interactions (Mideo and Reece 2012; Doherty et al. 2022) and thus were the focus of our analyses.

For comparisons of infection strategies (e.g., mantle lures, conglutinates, or broadcast), we tested assumptions of normality and homogeneity of variances using Shapiro–Wilk and Levene's tests, respectively. The Shapiro–Wilk test indicated that host specificity was normally distributed in the broadcast (*W* = 0.93, *p* = 0.27) and mantle lure (*W* = 0.97, *p* = 0.24) groups, but not in the conglutinate group (*W* = 0.85, *p* < 0.001). Variances were approximately equal (*F* = 2.916, *p* = 0.058). Due to the violation of normality in the conglutinate group, we applied a non‐parametric Kruskal–Wallis test to assess differences in host specificity across infection strategies. When significant, we conducted post hoc pairwise comparisons using Dunn's test (*DunnTest*) from the R package *FSA*. We had overlapping data for 102 species. A corresponding phylogenetic ANOVA was restricted to 41 species due to the availability of overlapping phylogenetic, life history, and specificity data.

To examine the relationship between mussel fecundity and host specificity, we first employed a linear regression model (R function lm) using fecundity as a continuous predictor and specificity as the response variable.

We also performed a phylogenetic generalized least squares (PGLS) analysis to evaluate the relationship while accounting for shared evolutionary history. The PGLS analysis was restricted to a subset of 24 species, compared to the 65 species included in the standard regression models. All statistical analyses were conducted in R version [4.3.2] using base functions and the stats, car, and caper packages.

### 2.6 Mussel–Fish Life‐History Association

For each mussel life‐history strategy, we tallied all fish species documented as hosts and calculated the proportion of hosts belonging to each fish life‐history category. These counts were then summarized in a contingency table for analysis. We used a Pearson's chi‐square test (function: *chisq.test*) to assess whether mussel life‐history strategies were associated with non‐random use of fish life‐history strategies. To identify which specific pairings contributed most to deviations from independence, we examined standardized Pearson residuals. Positive residuals indicate mussel–fish combinations that occur more frequently than expected under independence, whereas negative residuals indicate under‐represented combinations. To test for differences among specific life‐history category pairs, we conducted post hoc pairwise chi‐square tests using the function *pairwiseNominalIndependence*.

## 3 Results

### 3.1 Range and Variation in Host Specificity

**FIGURE 3:**
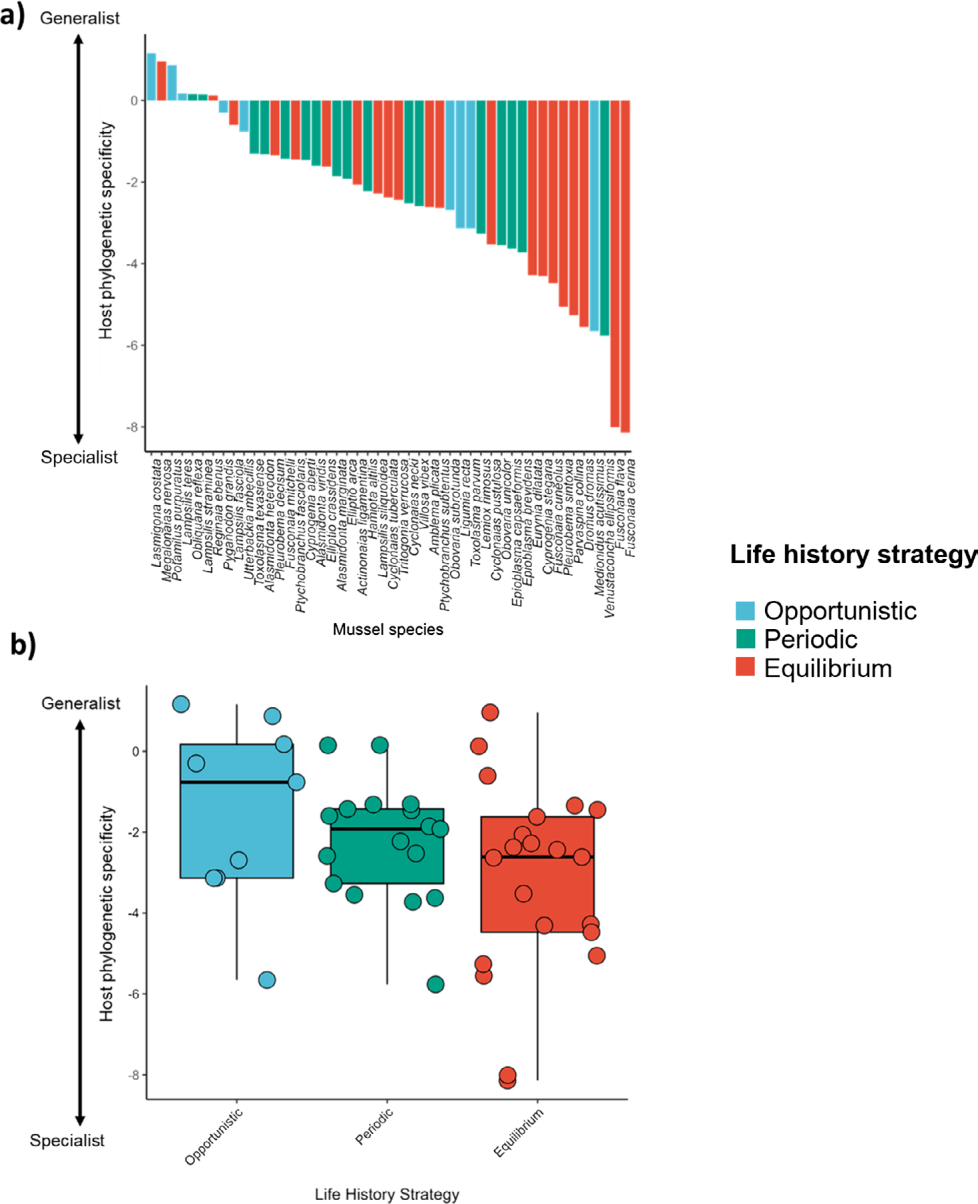
Relationship between host phylogenetic specificity and mussel life history strategies. (a) Host phylogenetic specificity (*y*‐axis) for each mussel species (*x*‐axis), colored by life history strategy: Opportunistic (blue), periodic (green), and equilibrium (red). Values closer to zero indicate broader host use, while more negative values reflect higher specificity. (b) Boxplot of host phylogenetic specificity grouped by mussel life history strategy, with individual species overlaid as points. The horizontal line within each box represents the median, box edges indicate the interquartile range (IQR), and whiskers extend to 1.5× the IQR.

Host specificity scores varied widely among North American freshwater mussel species. Among the 164 species analyzed, the number of reported host fish species ranged from 1 to 17. Phylogenetic host specificity scores spanned from −13.43 in *Theliderma cylindrica* , indicating extreme phylogenetic specificity, to +1.15 in *Lasmigona costata* , which exhibited phylogenetic generalism. The mean specificity score across species was −1.91 ± 2.13 (Figure 3a).

### 3.2 Life History Strategy and Host Specificity

Host specificity did not differ significantly among mussels classified into the three life history strategies (opportunistic, periodic, and equilibrium) based on a one‐way ANOVA (*F*_2,44_ = 2.18, *p* = 0.125, Figure 3b) or the phylogenetic ANOVA (*F*_2,41_ = 2.43, *p* = 0.137). Mean host specificity scores were −1.52 ± 2.29 for opportunistic species, −2.27 ± 1.51 for periodic species, and −3.18 ± 2.38 for equilibrium species.

Overall, SES MPD values were highly correlated among SIM9, SIM1 (equiprobable; sample.pool), and SIM3 (column sums fixed; richness), with Pearson correlation coefficients ranging from 0.96 to 0.997 (Table S3). In contrast, correlations between SIM2 (row sums fixed; frequency) and the other models were moderate (*r* = 0.52–0.54), indicating that controlling for mussel host breadth reduces but does not eliminate the observed phylogenetic structure (Table S3, Figure S1).

These results demonstrate that the observed clustering of mussel hosts is robust to the choice of null model, particularly when host richness and host sampling intensity are accounted for (SIM9, SIM3, and SIM1). The moderate difference observed with SIM2 suggests that variation in host breadth among mussels contributes partially to the patterns of clustering but does not drive the overall signal.

### 3.3 Traits and Phylogenetic Signal

Pagel's λ, which indicated significant phylogenetic structure for all three traits (λ > 0.6, *p* < 0.05). Phylogenetic signal was also detected in host specificity scores, as indicated by a moderate but significant Pagel's λ (λ = 0.32, *p* < 0.01), suggesting that closely related mussel species tend to exhibit more similar patterns of host use than expected by chance (Figure S2).

### 3.4 Mussel Traits Related to Reproduction and Parasitism Predict Specificity

**FIGURE 4:**
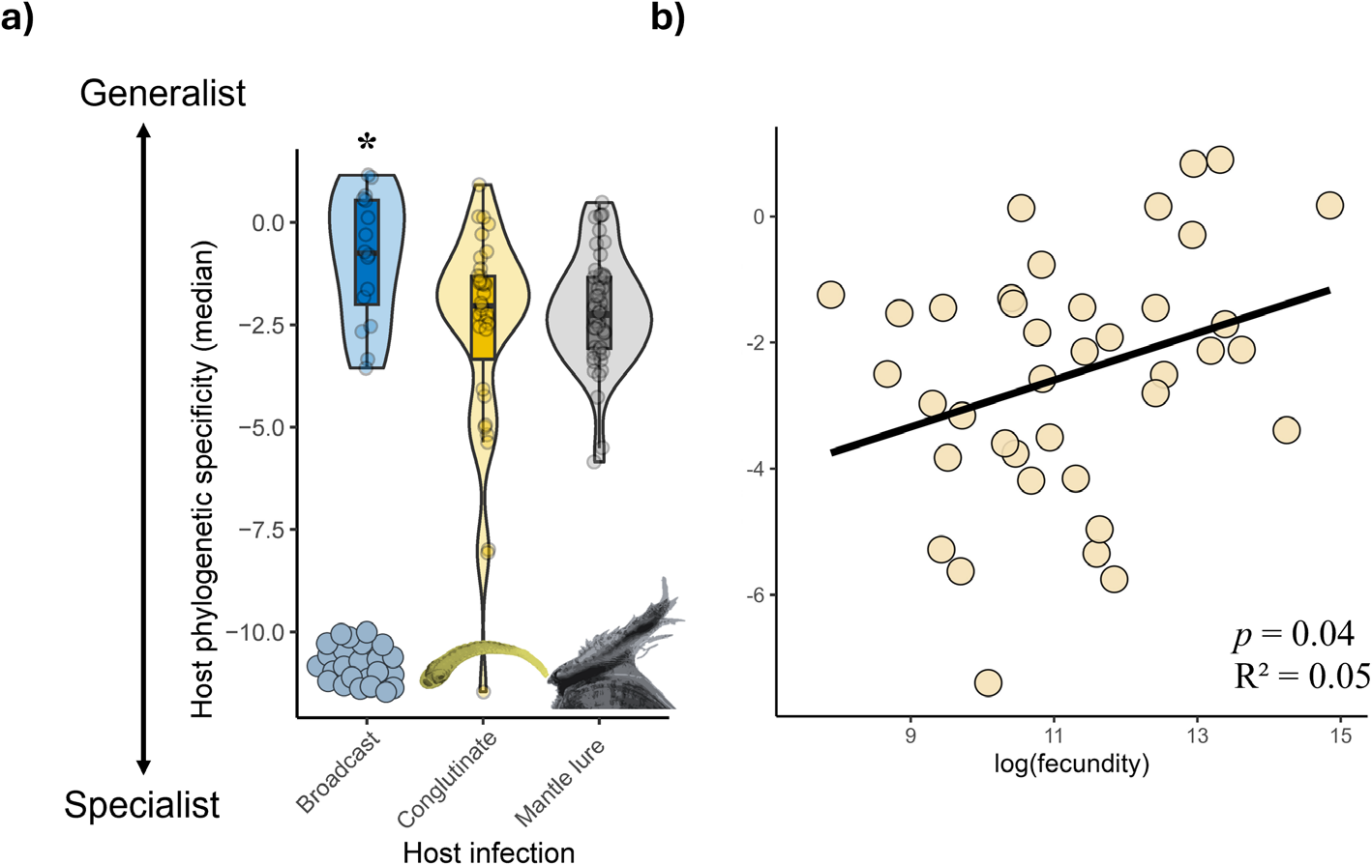
Relationships between reproductive traits and host phylogenetic specificity in freshwater mussels. (a) Violin plot showing host phylogenetic specificity across three host infection strategies: Conglutinate, broadcast, and mantle lure. The horizontal line within each box represents the median, box edges indicate the interquartile range (IQR), and whiskers extend to 1.5× the IQR. Lower values indicate higher host specificity. Illustrations below each category depict representative larval release strategies. Asterisk denotes significance. (b) Scatterplot showing the relationship between fecundity (log‐transformed for visual clarity) and host phylogenetic specificity for each mussel phylogenetic tribe (Pfeiffer et al. 2019). Each point represents a mussel species.

Host specificity differed significantly among host infection strategies (Kruskal–Wallis *χ* ^2^ = 7.58, df = 2, *p* = 0.023, Figure 4a). Post hoc Dunn's test indicated significant differences in host specificity between broadcast spawning mussels and those employing other host infection strategies. Specifically, broadcast species exhibited significantly different levels of host specificity compared to both conglutinate (*Z* = 2.42, *p* = 0.047) and mantle lure users (*Z* = 2.66, *p* = 0.023). In contrast, no significant difference in host specificity was observed between mussels utilizing conglutinate and mantle lure strategies (*Z* = 0.14, *p* = 1.0). When accounting for phylogeny and using a much smaller sample size, we found no differences among infection strategies (*F*_3,41_ = 1.15, *p* = 0.43, Figure S3). Fecundity was also a significant predictor of specificity. A linear regression across all species indicated a positive relationship between fecundity and host specificity (*F*_1,63_ = 4.64, *p* = 0.04, *R* ^2^ = 0.05, Figure 4b). When a phylogenetic correction was applied via PGLS, the relationship between fecundity and host specificity remained statistically significant (*F*_1,22_ = 4.52, *p* = 0.04, *R* ^2^ = 0.13, Figure S4).

### 3.5 Life History Strategy and Host–Fish Compatibility Patterns

**FIGURE 5:**
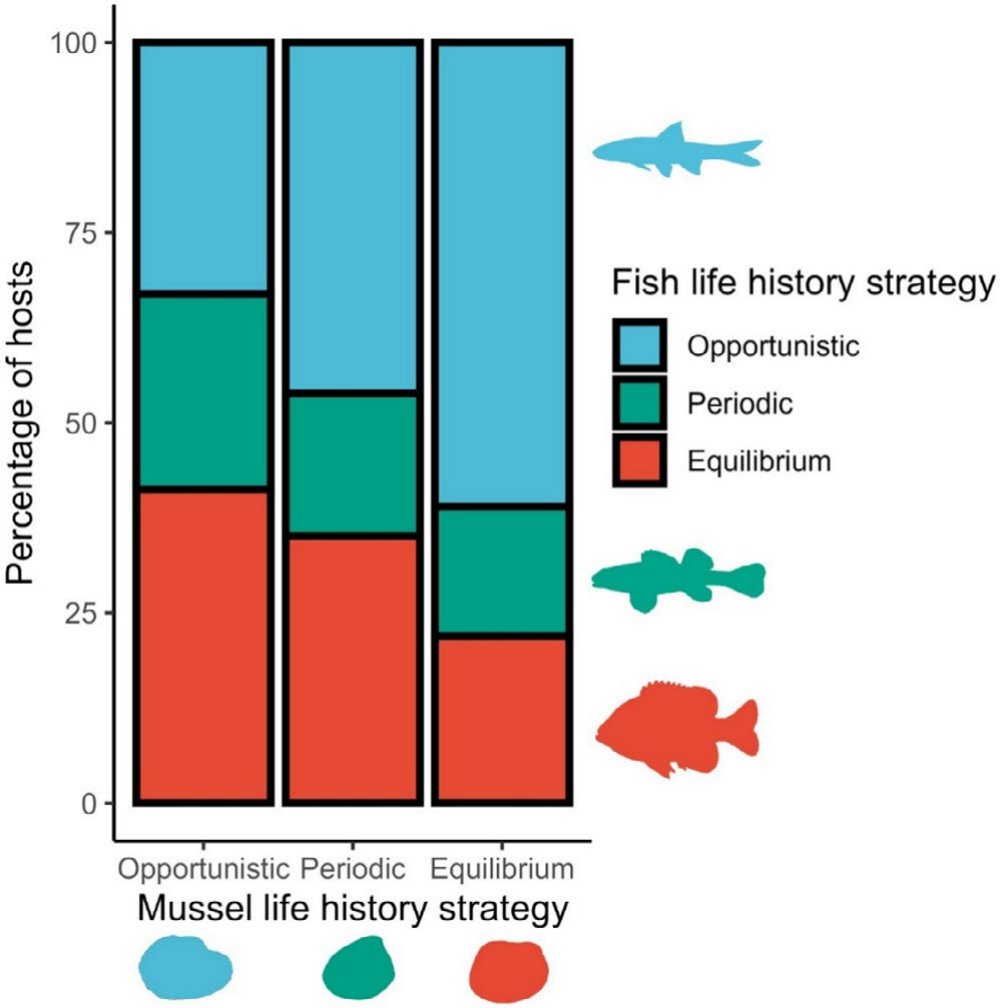
Proportion of host fish life history strategies used by mussel species with different life history strategies (opportunistic, periodic, equilibrium). Mussel life history strategies are shown on the *x*‐axis, and the *y*‐axis represents the percentage of host species grouped by fish life history strategy (color‐coded: Opportunistic = blue, periodic = green, equilibrium = red). Includes all mussel species with known host relationships and life history traits and strategies, including those inferred from congeners.

We analyzed 1170 mussel–fish host interactions, encompassing 164 mussel and 239 fish species, to examine life history strategy pairings. Equilibrium mussels used opportunistic fishes most often (60.1%) but also used equilibrium (22.0%) and periodic (17.1%) fishes. Mussels with a periodic life history strategy primarily used opportunistic (46.1%) and equilibrium (35.1%) fish species, while a smaller proportion of interactions were with periodic fish species (18.8%). Opportunistic mussels most frequently used equilibrium fishes (41.0%), followed by opportunistic (33.0%), and periodic (25.7%) fishes (Figure 5).

Mussel–fish life‐history associations deviated significantly from random expectation (*χ* ^2^ = 50.26, df = 4, *p* < 0.01). Standardized residuals showed that equilibrium mussels used opportunistic fishes more often than expected (+6.02) and equilibrium fishes less often (−5.16), while opportunistic mussels overused equilibrium fishes (+3.39) and underused opportunistic fishes (−5.22). Periodic mussels exhibited weaker but similar patterns, overusing equilibrium fishes (+2.16) and underusing opportunistic fishes (−1.52). Post hoc pairwise comparisons confirmed significant differences among all three mussel strategies in their patterns of fish‐strategy use (all adjusted *p* < 0.01).

## 4 Discussion

Our study synthesizes publicly available data on host phylogenetic specificity, life history traits, and host utilization to explore host–parasite dynamics in North American freshwater mussels (Unionidae), a diverse group of aquatic invertebrates that has been largely overlooked in parasitology, despite their obligate parasitic larval stage. We present the first comprehensive analysis of host phylogenetic specificity within unionids, revealing distinct patterns that connect the life history strategies of mussels and their fish hosts. Our findings indicate that host specificity is phylogenetically conserved, with closely related mussel species exhibiting similar patterns of host phylogenetic specificity. Patterns in mussel life history strategies show a general directional tendency in host use, with opportunistic species tending toward more generalist host use, periodic species exhibiting intermediate patterns, and equilibrium species showing relatively more specialized host use. Life history traits such as fecundity and host infection strategy are broadly aligned with these patterns, with species characterized by higher reproductive output or less targeted infection modes tending toward broader host use, and species with lower fecundity or more specialized infection strategies tending toward narrower host use. Across 169 mussel and 279 fish species, equilibrium mussels more frequently use short‐lived, fast‐developing opportunistic fish hosts, whereas opportunistic mussels preferentially use longer‐lived equilibrium fish hosts. This underscores important ecological and evolutionary patterns that shape host–parasite relationships in freshwater mussels.

We show that freshwater mussels exhibit a high degree of host specialization. On average, mussels used more phylogenetically clustered hosts than expected by chance. Some species, such as *Theliderma cylindrica* , exhibited highly clustered host use, while others, like *Lasmigona costata* , were phylogenetically overdispersed. Although *L. costata* has 14 reported hosts across a broad geographic range, these records may reflect physiological compatibility in laboratory settings rather than consistent use in the wild. This highlights the need for continued field‐based validation of host associations to distinguish between potential and ecologically relevant hosts (Hopper et al. 2025). However, employing phylogenetic specificity scores mitigates biases related to uneven research efforts, as these scores are less affected by the sheer number of published host records (Poulin and Mouillot 2005). We also found a strong relationship between phylogenetic host specificity and phylogenetic signal, supporting the idea that host use is shaped by evolutionary history. Previous studies on freshwater mussels have noted phylogenetic patterns based on host counts (Hewitt et al. 2019), our study advances our understanding of the influence of evolutionary history on mussel‐host relationships by quantifying host specificity using a phylogenetic framework. Two parasitic species may have a similar number of hosts yet differ substantially in how closely related those hosts are. By incorporating phylogenetic specificity into our assessment, we can capture deeper evolutionary patterns that extend beyond host diversity. This approach is especially important in regions of high biodiversity and endemism, such as the southeastern United States, a hotspot for fish and mussel biodiversity (Williams et al. 2008). In these systems, mussels may use different but closely related fish species across their range (e.g., *Micropterus coosae* and *M. warriorensis*, Baker et al. 2013), reflecting fine‐scale ecological and evolutionary processes. Growing evidence also suggests that populations of the same mussel species may rely on different hosts in different watersheds (Hopper et al. 2025). Understanding variation in host use is important not only for predicting the potential for host switching, but also for advancing our understanding of the ecological and evolutionary dynamics shaping mussel–host interactions.

Life history strategy may influence the degree of host specificity in freshwater mussels. While we did not detect statistically significant differences in host specificity across mussel life history strategies, we observed a directional trend. Opportunistic mussels (short‐lived, fast‐growing, and highly fecund) tended to use more phylogenetically diverse hosts than equilibrium mussels (long‐lived, slow‐growing, and low fecundity). Periodic strategists showed intermediate patterns. These patterns may arise from trade‐offs in resource allocation: fast‐paced species tend to invest less in mechanisms that enable fine‐tuned host targeting and more in high reproductive output, making broader host use an advantageous strategy (Doherty et al. 2022). Conversely, equilibrium mussels, generally found in more stable environments (Randklev et al. 2019; Haag 2012), may be under stronger pressure to identify high‐quality, predictable hosts, where stable coevolutionary relationships can develop, favoring the evolution of narrow host ranges and specialized infection strategies. These results suggest that mussel life history traits can shape the evolution of host specificity.

Infection strategy was associated with host specificity in freshwater mussel species. Species that use broadcast spawning, which involves releasing larvae freely into the water (Hewitt et al. 2021; Barnhart et al. 2008), exhibited significantly lower specificity compared to species that use more targeted strategies like mantle lures or conglutinates. While broadcasting is a passive infection strategy, it also increases the encounter chances with a broader suite of hosts. This pattern mirrors findings in other parasitic organisms, where transmission mode is closely linked to host specificity. Strategies that enhance host encounters tend to involve a broader host range but also depend more on host availability and susceptibility (Pedersen et al. 2005). While the phylogenetic ANOVA did not reveal significant differences among infection strategies, our phylogenetically constrained analysis was limited to only half of the species for which we had data in the non‐phylogenetic analysis. This restriction reduces our ability to detect broader patterns across the entire phylogenetic tree. Although broad phylogenetic patterns likely reflect deep‐time coevolution and conserved ecological niches (Wiens et al. 2010), this does not diminish the ecological relevance of infection mode as a mechanism to target species with varying degrees of phylogenetic relatedness.

Reproductive output, measured as fecundity, was positively associated with broader phylogenetic host use, and this relationship remained significant after accounting for shared evolutionary history. This suggests that highly fecund species tend to exploit a more phylogenetically diverse set of hosts, and that this pattern is not solely driven by phylogenetic relatedness among species. One potential mechanism is that high fecundity increases the likelihood of successful infection across a wider range of hosts. Similar links between reproductive output and generalist strategies have been reported in other parasitic organisms (Doherty et al. 2022), suggesting a common ecological trade‐off in which increased propagule production compensates for reduced host specialization. At the same time, the relatively weak effect size underscores that host use in freshwater mussels is shaped by multiple interacting factors, including evolutionary history and host availability (Neemuchwala et al. 2023; Hornbach et al. 2024), with fecundity acting as one of several traits that may facilitate broader host exploitation.

Life history traits such as reproductive rate, growth patterns, and dispersal capacity influence how species occupy niches, respond to habitat variability, and adapt to biotic interactions (Albaladejo‐Robles et al. 2023; Boyko et al. 2023; Weil et al. 2022). Within this context, examining host–parasite associations’ trilateral equilibrium–periodic–opportunistic (E–P–O) life history framework (Haag 2012; Winemiller and Rose 1992) reveals directional tendencies in host use. Our findings show that equilibrium mussels, with slower development and stronger competitive ability, primarily parasitize opportunistic fishes, which mature quickly, reproduce rapidly, and have faster generation times (Xiang et al. 2021; Perkin et al. 2017; Zhu et al. 2025). Conversely, opportunistic mussels tend to rely on equilibrium fishes, while periodic mussels show intermediate patterns. These patterns are consistent with dynamics along the evolutionary pace‐of‐life continuum (Ricklefs and Wikelski 2002), where fast‐paced hosts often invest less in immune defenses (Agnew and Koella 1999; Sears et al. 2015). Due to their rapid generation times and high population turnover, opportunistic fishes continuously produce immunologically naive individuals, increasing both temporal host availability and overall susceptibility to parasitism. While fishes can develop adaptive immunity following exposure to glochidia (Rock et al. 2022; Barnhart et al. 2008), this constant influx of unexposed hosts maintains a vulnerable population, making opportunistic fishes especially attractive to highly competitive equilibrium mussels that benefit from specializing on more susceptible hosts. In contrast, opportunistic mussels may compensate for lower competitive ability and broader host use by using slower‐growing, better‐defended hosts, relying on high reproductive output to increase infection success.

Applying a life history framework to host‐mussel interactions creates a useful perspective for generating hypotheses about how host specificity evolves and persists and offers a foundation for future studies as we continue to gather data on host use, infection rates, and freshwater mussels and fish traits. While our data do not provide definitive evidence for these relationships, the observed mismatches in life history strategies and differences in host specificity suggest that host use in mussels may reflect evolutionary trade‐offs involving host defenses, competitive interactions, and parasite specialization. Integrating trait‐ and phylogeny‐based approaches, as commonly used in parasitology, may help clarify the ecological and evolutionary drivers of complex mussel life cycles. This perspective is especially important for conservation, as unionid mussels are highly host‐dependent and among the most imperiled groups in North America (Modesto et al. 2018; Haag and Williams 2014). Considering life history associations, such as equilibrium mussels relying on opportunistic fishes, or vice versa, emphasizes the need to consider both host and parasite traits in conservation planning. More broadly, conserving these interactions is critical for maintaining freshwater biodiversity and ecosystem function (Bascompte and Jordano 2007; Stachowicz 2001).

The patterns we observed likely reflect broader ecological and evolutionary principles common to many parasite taxa. Across parasites, host use is constrained by two fundamental factors: the probability of encountering a host and compatibility with that host (Combes 2001). Both encounter and compatibility are mediated by interacting parasite and host traits, including parasite reproductive output and generation time, as well as host lifespan, demography, and population stability (Koella et al. 1998; Agnew and Koella 1999). Life‐history frameworks link these dimensions by integrating traits of both parasites and hosts. In unionid mussels, these constraints can be directly quantified: fecundity and infection strategy shape encounter probability, whereas host phylogenetic relatedness and longevity provide proxies for compatibility. Life‐history trade‐offs potentially structure host specificity across a wide range of parasite taxa, with increased specialization often associated with higher performance on a restricted set of hosts but reduced transmission opportunities, while generalism trades per‐host efficiency for broader encounter potential (Futuyma and Moreno 1988; Leggett et al. 2013; Doherty et al. 2022). The convergence of these patterns suggests that trade‐offs between specialization and generalism are tightly linked to life‐history and reproductive traits, and that the relationships we identify between host use and life‐history trait variation may extend to other parasitic taxa in which encounter rates and compatibility are shaped by analogous ecological and evolutionary constraints.

## Author Contributions

**Irene Sánchez González:** conceptualization (lead), data curation (lead), formal analysis (lead), investigation (lead), methodology (lead), visualization (lead), writing – original draft (lead). **Andrew W. Park:** conceptualization (supporting), formal analysis (supporting), methodology (supporting), writing – review and editing (equal). **Krista A. Capps:** conceptualization (equal), funding acquisition (lead), resources (lead), supervision (lead), visualization (supporting), writing – review and editing (equal).

## Funding

This work was supported by the National Science Foundation Division of Environmental Biology (1941555).

## Conflicts of Interest

The authors declare no conflicts of interest.

## Supporting information


**Figure S1:** Standarized effect sizes of mean phylogenetic distance (SES MPD) for host specificity under four null model assumptions. Violin plots show the distribution of SES MPD values for SIM1 (equiprobable), SIM2 (row‑fixed), SIM3 (column‑fixed), and SIM9 (fixed–fixed) null models. Boxplots indicate medians and interquartile ranges, with points representing individual species. More negative SES MPD values indicate greater phylogenetic clustering of host use relative to null expectations.
**Figure S2:** Phylogenetic tree of freshwater mussel species with trait data visualized at the tips: left shows fecundity, center shows host specificity (log‐transformed), and right displays species names for reference.
**Figure S3:** Specificity (MPD. Obs Z) of freshwater mussel lineages across host infection strategies. Boxplots summarize the distribution of specificity for broadcast, conglutinate, and mantle lure infections, with points representing individual species. Colors indicate mussel tribe (Amblemini, Anodontini, Lampsilini, Pleurobemini, Quadrulini). Lower specificity values indicate greater phylogenetic clustering of host use.
**Figure S4:** Scatterplot showing the relationship between fecundity (log‐transformed for visual clarity) and host phylogenetic specificity using a Phylogenetic Generalized Least Square model. Each point represents a mussel species included in the model.
**Table S1:** List of fish host and their assigned life history strategy.
**Table S2:** List of mussel species and their assigned life history strategy.
**Table S3:** Pairwise Pearson correlations of standardized effect sizes of mean pairwise phylogenetic distance (SES MPD) among four null models of mussel host specificity (SIM1, SIM2, SIM3, SIM9). High correlations indicate that observed patterns of host phylogenetic clustering are robust to null model choice, whereas moderate correlations suggest sensitivity to specific constraints, such as mussel host breadth (SIM2).

## Data Availability

Data available from the OSF repository: https://osf.io/hqwyd/overview?view_only=a3d94c7b158d40378b294ab35008f577.
