# Supplementary material for: Trait‐Based Drivers of Host Specificity in Freshwater Mussel–Fish Parasitic Interactions
Source: Ecol Evol. 2026 Jul 28;16(8):e74097. doi: 10.1002/ece3.74097 (PMC13415976; doi:10.1002/ece3.74097)
Supplement: Supplementary file 1 — Figure S1: Standarized effect sizes of mean phylogenetic distance (SES MPD) for host specificity under four null model assumptions. Violin plots show the distribution of SES MPD values for SIM1 (equiprobable), SIM2 (row‑fixed), SIM3 (column‑fixed), and SIM9 (fixed–fixed) null models. Boxplots indicate medians and interquartile ranges, with points representing individual species. More negative SES MPD values indicate greater phylogenetic clustering of host use relative to null expectations. Figure S2: Phylogenetic tree of freshwater mussel species with trait data visualized at the tips: left shows fecundity, center shows host specificity (log‐transformed), and right displays species names for reference. Figure S3: Specificity (MPD. Obs Z) of freshwater mussel lineages across host infection strategies. Boxplots summarize the distribution of specificity for broadcast, conglutinate, and mantle lure infections, with points representing individual species. Colors indicate mussel tribe (Amblemini, Anodontini, Lampsilini, Pleurobemini, Quadrulini). Lower specificity values indicate greater phylogenetic clustering of host use. Figure S4: Scatterplot showing the relationship between fecundity (log‐transformed for visual clarity) and host phylogenetic specificity using a Phylogenetic Generalized Least Square model. Each point represents a mussel species included in the model. Table S1: List of fish host and their assigned life history strategy. Table S2: List of mussel species and their assigned life history strategy. Table S3: Pairwise Pearson correlations of standardized effect sizes of mean pairwise phylogenetic distance (SES MPD) among four null models of mussel host specificity (SIM1, SIM2, SIM3, SIM9). High correlations indicate that observed patterns of host phylogenetic clustering are robust to null model choice, whereas moderate correlations suggest sensitivity to specific constraints, such as mussel host breadth (SIM2). [file ECE3-16-e74097-s001.docx]

**Trait-based drivers of host specificity in freshwater mussel–fish parasitic interactions**

Irene Sánchez González^1*^, Andrew W. Park^1^ and Krista A. Capps^1^

Table S1. List of fish host and their assigned life history strategy.

| **Fish species** | **Life history strategy** |
| --- | --- |
| *Acipenser fulvescens* | Equilibrium |
| Acipenser oxyrinchus | Equilibrium |
| *Alburnops baileyi* | Opportunistic |
| *Alburnops blennius* | Opportunistic |
| *Alburnops chalybaeus* | Opportunistic |
| *Alburnops petersoni* | Opportunistic |
| *Alburnops texanus* | Opportunistic |
| *Alosa alabamae* | Periodic |
| *Alosa chrysochloris* | Periodic |
| *Alosa pseudoharengus* | Periodic |
| *Ambloplites ariommus* | Equilibrium |
| *Ambloplites rupestris* | Equilibrium |
| *Ameiurus brunneus* | Equilibrium |
| *Ameiurus melas* | Equilibrium |
| *Ameiurus natalis* | Equilibrium |
| *Ameiurus nebulosus* | Equilibrium |
| *Amia calva* | Periodic |
| *Ammocrypta beanii* | Opportunistic |
| *Ammocrypta meridiana* | Opportunistic |
| *Ammocrypta pellucida* | Opportunistic |
| *Anguilla rostrata* | Periodic |
| *Aphredoderus sayanus* | Periodic |
| *Aplodinotus grunniens* | Periodic |
| *Atractosteus spatula* | Periodic |
| *Campostoma anomalum* | Opportunistic |
| *Campostoma oligolepis* | Opportunistic |
| *Carpiodes carpio* | Periodic |
| *Carpiodes cyprinus* | Periodic |
| *Catostomus commersonii* | Periodic |
| *Chrosomus eos* | Opportunistic |
| *Chrosomus erythrogaster* | Opportunistic |
| *Chrosomus oreas* | Opportunistic |
| *Clinostomus elongatus* | Opportunistic |
| *Clinostomus funduloides* | Opportunistic |
| *Coccotis coccogenis* | Opportunistic |
| *Cottus asper* | Equilibrium |
| *Cottus baileyi* | Equilibrium |
| *Cottus bairdii* | Equilibrium |
| *Cottus carolinae* | Equilibrium |
| *Cottus cognatus* | Equilibrium |
| *Cottus marginatus* | Equilibrium |
| *Culaea inconstans* | Opportunistic |
| *Cyprinella analostana* | Opportunistic |
| *Cyprinella callistia* | Opportunistic |
| *Cyprinella galactura* | Opportunistic |
| *Cyprinella lutrensis* | Opportunistic |
| *Cyprinella nivea* | Opportunistic |
| *Cyprinella spiloptera* | Opportunistic |
| *Cyprinella trichroistia* | Opportunistic |
| *Cyprinella venusta* | Opportunistic |
| *Cyprinella whipplei* | Opportunistic |
| *Cyprinus carpio* | Periodic |
| *Dorosoma cepedianum* | Periodic |
| *Dorosoma petenense* | Periodic |
| *Ericymba amplamala* | Opportunistic |
| *Ericymba buccata* | Opportunistic |
| *Ericymba dorsalis* | Opportunistic |
| *Erimystax dissimilis* | Opportunistic |
| *Erimystax insignis* | Opportunistic |
| *Erimyzon oblongus* | Opportunistic |
| *Esox lucius* | Periodic |
| *Etheostoma artesiae* | Opportunistic |
| *Etheostoma asprigene* | Opportunistic |
| *Etheostoma baileyi* | Opportunistic |
| *Etheostoma bellator* | Opportunistic |
| *Etheostoma blennioides* | Opportunistic |
| *Etheostoma caeruleum* | Opportunistic |
| *Etheostoma camurum* | Opportunistic |
| *Etheostoma coosae* | Opportunistic |
| *Etheostoma douglasi* | Opportunistic |
| *Etheostoma edwini* | Opportunistic |
| *Etheostoma exile* | Opportunistic |
| *Etheostoma flabellare* | Opportunistic |
| *Etheostoma fusiforme* | Opportunistic |
| *Etheostoma inscriptum* | Opportunistic |
| *Etheostoma jordani* | Opportunistic |
| *Etheostoma juliae* | Opportunistic |
| *Etheostoma kennicotti* | Opportunistic |
| *Etheostoma lepidum* | Opportunistic |
| *Etheostoma maculatum* | Opportunistic |
| *Etheostoma nigrum* | Opportunistic |
| *Etheostoma obeyense* | Opportunistic |
| *Etheostoma olivaceum* | Opportunistic |
| *Etheostoma olmstedi* | Opportunistic |
| *Etheostoma rufilineatum* | Opportunistic |
| *Etheostoma rupestre* | Opportunistic |
| *Etheostoma sagitta* | Opportunistic |
| *Etheostoma simoterum* | Opportunistic |
| *Etheostoma spectabile* | Opportunistic |
| *Etheostoma stigmaeum* | Opportunistic |
| *Etheostoma swaini* | Opportunistic |
| *Etheostoma tippecanoe* | Opportunistic |
| *Etheostoma virgatum* | Opportunistic |
| *Etheostoma vulneratum* | Opportunistic |
| *Etheostoma whipplei* | Opportunistic |
| *Etheostoma zonale* | Opportunistic |
| *Fundulus catenatus* | Opportunistic |
| *Fundulus diaphanus* | Opportunistic |
| *Fundulus notatus* | Opportunistic |
| *Fundulus olivaceus* | Opportunistic |
| *Gambusia affinis* | Opportunistic |
| *Gambusia holbrooki* | Opportunistic |
| *Gasterosteus aculeatus* | Opportunistic |
| *Herichthys cyanoguttatus* | Periodic |
| *Hiodon alosoides* | Equilibrium |
| *Hudsonius altipinnis* | Opportunistic |
| *Hudsonius hudsonius* | Opportunistic |
| *Hybognathus hankinsoni* | Opportunistic |
| *Hybognathus nuchalis* | Opportunistic |
| *Hybopsis amblops* | Opportunistic |
| *Hybopsis winchelli* | Opportunistic |
| *Hydrophlox chiliticus* | Opportunistic |
| *Hydrophlox lutipinnis* | Opportunistic |
| *Hydrophlox rubricroceus* | Opportunistic |
| *Hypentelium etowanum* | Equilibrium |
| *Hypentelium nigricans* | Equilibrium |
| *Ictalurus furcatus* | Periodic |
| *Ictalurus punctatus* | Periodic |
| *Ictiobus bubalus* | Periodic |
| *Ictiobus cyprinellus* | Periodic |
| *Labidesthes sicculus* | Periodic |
| *Lepisosteus osseus* | Periodic |
| *Lepisosteus platostomus* | Periodic |
| *Lepisosteus platyrhincus* | Periodic |
| *Lepomis auritus* | Equilibrium |
| *Lepomis cyanellus* | Equilibrium |
| *Lepomis gibbosus* | Equilibrium |
| *Lepomis gulosus* | Equilibrium |
| *Lepomis humilis* | Equilibrium |
| *Lepomis macrochirus* | Equilibrium |
| *Lepomis megalotis* | Equilibrium |
| *Lepomis microlophus* | Equilibrium |
| *Lepomis punctatus* | Equilibrium |
| *Luxilus albeolus* | Opportunistic |
| *Luxilus cardinalis* | Opportunistic |
| *Luxilus chrysocephalus* | Opportunistic |
| *Luxilus cornutus* | Opportunistic |
| *Luxilus pilsbryi* | Opportunistic |
| *Luxilus zonatus* | Opportunistic |
| *Lythrurus ardens* | Opportunistic |
| *Lythrurus bellus* | Opportunistic |
| *Lythrurus fasciolaris* | Opportunistic |
| *Lythrurus umbratilis* | Opportunistic |
| *Lytrhrurus matutinus* | Opportunistic |
| *Macrhybopsis storeriana* | Opportunistic |
| *Margariscus margarita* | Opportunistic |
| *Micropterus cataractae* | Equilibrium |
| *Micropterus coosae* | Equilibrium |
| *Micropterus dolomieu* | Equilibrium |
| *Micropterus nigricans* | Equilibrium |
| *Micropterus punctulatus* | Equilibrium |
| *Micropterus treculii* | Equilibrium |
| *Miniellus alborus* | Opportunistic |
| *Miniellus ammophilus* | Opportunistic |
| *Miniellus boops* | Opportunistic |
| *Miniellus heterodon* | Opportunistic |
| *Miniellus longirostris* | Opportunistic |
| *Miniellus nubilus* | Opportunistic |
| *Miniellus stramineus* | Opportunistic |
| *Morone americana* | Periodic |
| *Morone chrysops* | Periodic |
| *Morone saxatilis* | Periodic |
| *Moxostoma anisurum* | Periodic |
| *Moxostoma congestum* | Periodic |
| *Moxostoma erythrurum* | Periodic |
| *Moxostoma lachneri* | Periodic |
| *Moxostoma macrolepidotum* | Periodic |
| *Moxostoma robustum* | Periodic |
| *Moxostoma rupiscartes* | Periodic |
| *Neogobius melanostomus* | Periodic |
| *Nocomis biguttatus* | Opportunistic |
| *Nocomis leptocephalus* | Opportunistic |
| *Nocomis micropogon* | Opportunistic |
| *Notemigonus crysoleucas* | Periodic |
| *Notropis ariommus* | Opportunistic |
| *Notropis atherinoides* | Opportunistic |
| *Notropis heterodon* | Opportunistic |
| *Notropis heterolepis* | Opportunistic |
| *Notropis percobromus* | Opportunistic |
| *Notropis photogenis* | Opportunistic |
| *Notropis rubellus* | Opportunistic |
| *Notropis stilbius* | Opportunistic |
| *Notropis telescopus* | Opportunistic |
| *Notropis xaenocephalus* | Opportunistic |
| *Noturus exilis* | Opportunistic |
| *Noturus flavus* | Opportunistic |
| *Noturus funebris* | Opportunistic |
| *Noturus gyrinus* | Opportunistic |
| *Noturus leptocanthus* | Opportunistic |
| *Oncorhynchus kisutch* | Equilibrium |
| *Oncorhynchus mykiss* | Equilibrium |
| *Oncoryhnchus clarkii* | Equilibrium |
| *Paranotropis leuciodus* | Opportunistic |
| *Paranotropis volucellus* | Opportunistic |
| *Perca flavescens* | Periodic |
| *Percina aurantiaca* | Periodic |
| *Percina burtoni* | Periodic |
| *Percina caprodes* | Periodic |
| *Percina copelandi* | Periodic |
| *Percina crypta* | Periodic |
| *Percina evides* | Periodic |
| *Percina kathae* | Periodic |
| *Percina maculata* | Periodic |
| *Percina nigrofasciata* | Opportunistic |
| *Percina palmaris* | Periodic |
| *Percina peltata* | Periodic |
| *Percina phoxocephala* | Periodic |
| *Percina roanoka* | Periodic |
| *Percina sciera* | Periodic |
| *Percina shumardi* | Periodic |
| *Percina vigil* | Periodic |
| *Phenacobius mirabilis* | Opportunistic |
| *Pimephales notatus* | Opportunistic |
| *Pimephales promelas* | Opportunistic |
| *Pimephales vigilax* | Opportunistic |
| *Poecilia reticulata* | Opportunistic |
| *Poecilia sphenops* | Opportunistic |
| *Pomoxis annularis* | Equilibrium |
| *Pomoxis nigromaculatus* | Equilibrium |
| *Pteronotropis grandipinnis* | Opportunistic |
| *Pteronotropis hypselopterus* | Opportunistic |
| *Pteronotropis signipinnis* | Opportunistic |
| *Pylodictis olivaris* | Equilibrium |
| *Rhinichthys atratulus* | Opportunistic |
| *Rhinichthys cataractae* | Opportunistic |
| *Rhinichthys obstusus* | Opportunistic |
| *Rhinichthys osculus* | Opportunistic |
| *Salmo salar* | Equilibrium |
| *Salmo trutta* | Equilibrium |
| *Salvelinus malma* | Equilibrium |
| *Sander canadensis* | Periodic |
| *Sander vitreus* | Periodic |
| *Scaphirhynchus platorynchus* | Periodic |
| *Semotilus atromaculatus* | Opportunistic |
| *Semotilus corporalis* | Opportunistic |
| *Umbra limi* | Opportunistic |

Table S2. List of mussel species and their assigned life history strategy.

| ***Mussel species*** | ***Life history strategy*** |
| --- | --- |
| *Actinonaias ligamentina* | *Equilibrium* |
| *Actinonaias pectorosa* | *Equilibrium* |
| *Alasmidonta arcula* | *Periodic* |
| *Alasmidonta atropurpurea* | *Periodic* |
| *Alasmidonta heterodon* | *Periodic* |
| *Alasmidonta marginata* | *Periodic* |
| *Alasmidonta raveneliana* | *Periodic* |
| *Alasmidonta undulata* | *Periodic* |
| *Alasmidonta varicosa* | *Periodic* |
| *Alasmidonta viridis* | *Periodic* |
| *Amblema neislerii* | *Equilibrium* |
| *Amblema plicata* | *Equilibrium* |
| *Anodonta californiensis* | *Opportunistic* |
| *Anodonta kennerlyi* | *Opportunistic* |
| *Anodonta oregonensis* | *Opportunistic* |
| *Arcidens confragosus* | *Opportunistic* |
| *Arcidens wheeleri* | *Opportunistic* |
| *Cambarunio iris* | *Opportunistic* |
| *Cambarunio nebulosa* | *Periodic* |
| *Cambarunio taeniatus* | *Opportunistic* |
| *Cyclonaias kieneriana* | *Equilibrium* |
| *Cyclonaias necki* | *Periodic* |
| *Cyclonaias nodulata* | *Equilibrium* |
| *Cyclonaias pustulosa* | *Equilibrium* |
| *Cyclonaias succissa* | *Equilibrium* |
| *Cyclonaias tuberculata* | *Equilibrium* |
| *Cyprogenia aberti* | *Periodic* |
| *Cyprogenia stegaria* | *Equilibrium* |
| *Cyrtonaias tampicoensis* | *Opportunistic* |
| *Dromus dromas* | *Equilibrium* |
| *Ellipsaria lineolata* | *Periodic* |
| *Elliptio arca* | *Periodic* |
| *Elliptio crassidens* | *Equilibrium* |
| *Elliptio fisheriana* | *Periodic* |
| *Elliptio hopetonensis* | *Periodic* |
| *Elliptio jayensis* | *Periodic* |
| *Elliptio pullata* | *Periodic* |
| *Elliptio shepardiana* | *Periodic* |
| *Elliptoideus sloatianus* | *Periodic* |
| *Epioblasma ahlstedti* | *Periodic* |
| *Epioblasma brevidens* | *Periodic* |
| *Epioblasma capsaeformis* | *Periodic* |
| *Epioblasma florentina* | *Periodic* |
| *Epioblasma obliquata* | *Periodic* |
| *Epioblasma penita* | *Periodic* |
| *Epioblasma rangiana* | *Periodic* |
| *Epioblasma triquetra* | *Periodic* |
| *Eurynia dilatata* | *Equilibrium* |
| *Fusconaia burkei* | *Equilibrium* |
| *Fusconaia cerina* | *Equilibrium* |
| *Fusconaia cor* | *Equilibrium* |
| *Fusconaia cuneolus* | *Equilibrium* |
| *Fusconaia escambia* | *Equilibrium* |
| *Fusconaia flava* | *Equilibrium* |
| *Fusconaia masoni* | *Equilibrium* |
| *Fusconaia mitchelli* | *Periodic* |
| *Fusconaia ozarkensis* | *Equilibrium* |
| *Glebula rotundata* | *Periodic* |
| *Hamiota altilis* | *Periodic* |
| *Hamiota perovalis* | *Periodic* |
| *Hamiota subangulata* | *Periodic* |
| *Lampsilis abrupta* | *Periodic* |
| *Lampsilis bracteata* | *Periodic* |
| *Lampsilis cardium* | *Periodic* |
| *Lampsilis dolabraeformis* | *Periodic* |
| *Lampsilis fasciola* | *Periodic* |
| *Lampsilis floridensis* | *Periodic* |
| *Lampsilis higginsii* | *Periodic* |
| *Lampsilis hydiana* | *Periodic* |
| *Lampsilis ornata* | *Opportunistic* |
| *Lampsilis ovata* | *Periodic* |
| *Lampsilis rafinesqueana* | *Periodic* |
| *Lampsilis reeveiana* | *Periodic* |
| *Lampsilis satura* | *Periodic* |
| *Lampsilis sietmani* | *Periodic* |
| *Lampsilis siliquoidea* | *Equilibrium* |
| *Lampsilis splendida* | *Periodic* |
| *Lampsilis straminea* | *Periodic* |
| *Lampsilis teres* | *Opportunistic* |
| *Lampsilis virescens* | *Periodic* |
| *Lasmigona complanata* | *Periodic* |
| *Lasmigona compressa* | *Periodic* |
| *Lasmigona costata* | *Periodic* |
| *Lasmigona decorata* | *Periodic* |
| *Lasmigona etowaensis* | *Periodic* |
| *Lasmigona holstonia* | *Periodic* |
| *Leaunio lienosus* | *Periodic* |
| *Leaunio ortmanni* | *Opportunistic* |
| *Leaunio vanuxemensis* | *Periodic* |
| *Lemiox rimosus* | *Periodic* |
| *Ligumia recta* | *Periodic* |
| *Medionidus acutissimus* | *Periodic* |
| *Medionidus conradicus* | *Periodic* |
| *Medionidus penicillatus* | *Periodic* |
| *Medionidus walkeri* | *Periodic* |
| *Megalonaias nervosa* | *Equilibrium* |
| *Obliquaria reflexa* | *Periodic* |
| *Obovaria olivaria* | *Periodic* |
| *Obovaria subrotunda* | *Periodic* |
| *Obovaria unicolor* | *Periodic* |
| *Paetulunio fabalis* | *Opportunistic* |
| *Parvaspina collina* | *Equilibrium* |
| *Parvaspina steinstansana* | *Equilibrium* |
| *Pegias fabula* | *Equilibrium* |
| *Plethobasus cyphyus* | *Equilibrium* |
| *Pleurobema clava* | *Equilibrium* |
| *Pleurobema cordatum* | *Equilibrium* |
| *Pleurobema decisum* | *Equilibrium* |
| *Pleurobema georgianum* | *Equilibrium* |
| *Pleurobema oviforme* | *Equilibrium* |
| *Pleurobema pyriforme* | *Equilibrium* |
| *Pleurobema riddellii* | *Equilibrium* |
| *Pleurobema rubellum* | *Equilibrium* |
| *Pleurobema rubrum* | *Equilibrium* |
| *Pleurobema sintoxia* | *Equilibrium* |
| *Pleurobema strodeanum* | *Equilibrium* |
| *Pleuronaia dolabelloides* | *Equilibrium* |
| *Popenaias. popeii* | *Periodic* |
| *Potamilus alatus* | *Opportunistic* |
| *Potamilus amphichaenus* | *Opportunistic* |
| *Potamilus capax* | *Opportunistic* |
| *Potamilus fragilis* | *Opportunistic* |
| *Potamilus inflatus* | *Opportunistic* |
| *Potamilus leptodon* | *Opportunistic* |
| *Potamilus metnecktayi* | *Opportunistic* |
| *Potamilus ohiensis* | *Opportunistic* |
| *Potamilus purpuratus* | *Opportunistic* |
| *Pseudodontoideus connasaugaensis* | *Opportunistic* |
| *Pseudodontoideus subvexus* | *Opportunistic* |
| *Ptychobranchus fasciolaris* | *Equilibrium* |
| *Ptychobranchus formanianus* | *Equilibrium* |
| *Ptychobranchus greenii* | *Equilibrium* |
| *Ptychobranchus jonesi* | *Equilibrium* |
| *Ptychobranchus occidentalis* | *Equilibrium* |
| *Ptychobranchus subtentus* | *Equilibrium* |
| *Pyganodon cataracta* | *Opportunistic* |
| *Pyganodon grandis* | *Opportunistic* |
| *Quadrula fragosa* | *Equilibrium* |
| *Quadrula quadrula* | *Equilibrium* |
| *Reginaia ebenus* | *Equilibrium* |
| *Sagittunio nasutus* | *Opportunistic* |
| *Sagittunio subrostratus* | *Opportunistic* |
| *Strophitus undulatus* | *Periodic* |
| *Theliderma cylindrica* | *Periodic* |
| *Theliderma intermedia* | *Equilibrium* |
| *Theliderma metanerva* | *Equilibrium* |
| *Toxolasma cylindrellus* | *Periodic* |
| *Toxolasma lividum* | *Periodic* |
| *Toxolasma parvum* | *Opportunistic* |
| *Toxolasma texasiense* | *Periodic* |
| *Tritogonia nobilis* | *Equilibrium* |
| *Tritogonia verrucosa* | *Equilibrium* |
| *Truncilla donaciformis* | *Opportunistic* |
| *Truncilla truncata* | *Opportunistic* |
| *Uniomerus tetralasmus* | *Opportunistic* |
| *Utterbackia imbecillis* | *Opportunistic* |
| *Utterbackia suborbiculata* | *Opportunistic* |
| *Utterbackiana implicata* | *Opportunistic* |
| *Venustaconcha constricta* | *Periodic* |
| *Venustaconcha ellipsiformis* | *Periodic* |
| *Venustaconcha trabilis* | *Periodic* |
| *Villosa delumbis* | *Periodic* |
| *Villosa vibex* | *Periodic* |
| *Villosa villosa* | *Periodic* |

Table S3. Pairwise Pearson correlations of standardized effect sizes of mean pairwise phylogenetic distance (SES MPD) among four null models of mussel host specificity (SIM1, SIM2, SIM3, SIM9). High correlations indicate that observed patterns of host phylogenetic clustering are robust to null model choice, whereas moderate correlations suggest sensitivity to specific constraints, such as mussel host breadth (SIM2).

|  | **SIM8** | **SIM2** | **SIM3** | **SIM1** |
| --- | --- | --- | --- | --- |
| **SIM9** | 1.00 | 0.56 | 0.97 | 0.97 |
| **SIM2** | 0.56 | 1.00 | 0.53 | 0.53 |
| **SIM3** | 0.97 | 0.53 | 1.00 | 1.00 |
| **SIM1** | 0.97 | 0.53 | 1.00 | 1.00 |


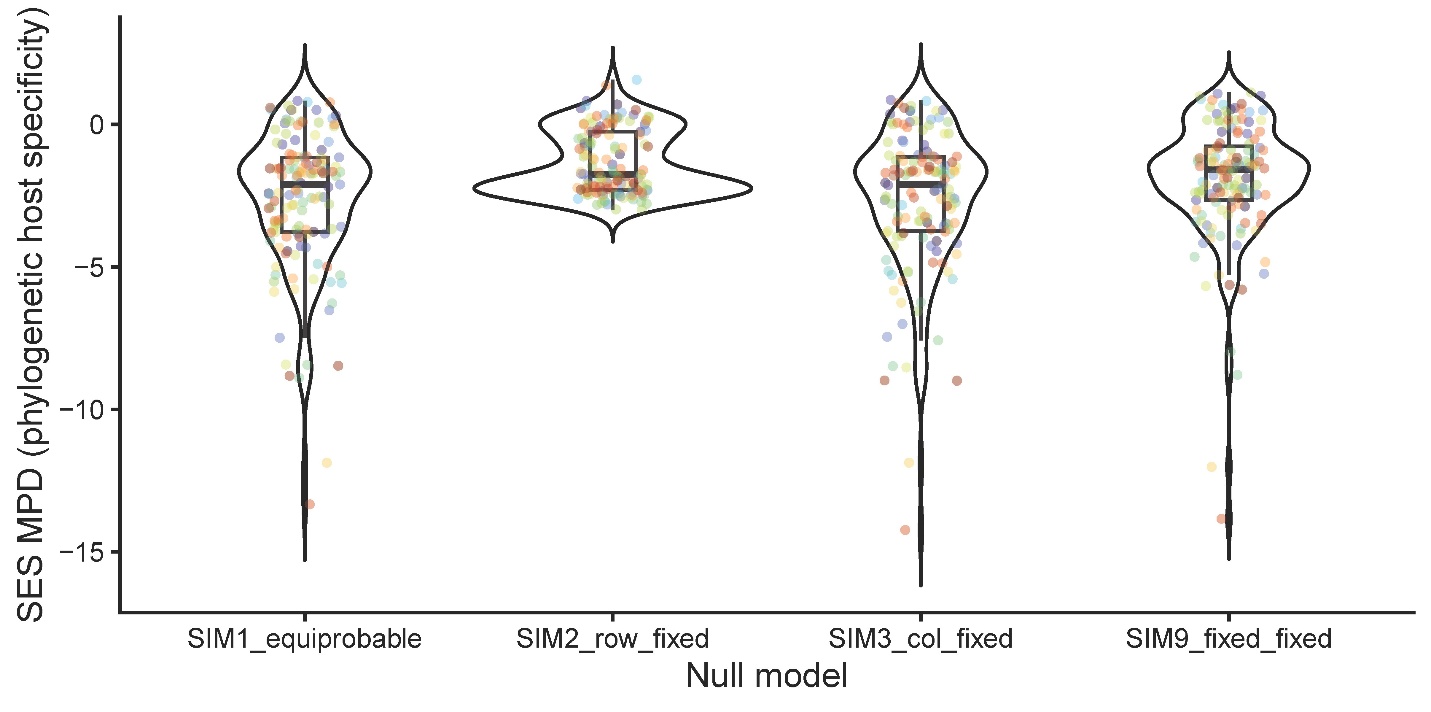


Figure S1. Standarized effect sizes of mean phylogenetic distance (SES MPD) for host specificity under four null model assumptions. Violin plots show the distribution of SES MPD values for SIM1 (equiprobable), SIM2 (row‑fixed), SIM3 (column‑fixed), and SIM9 (fixed–fixed) null models. Boxplots indicate medians and interquartile ranges, with points representing individual species. More negative SES MPD values indicate greater phylogenetic clustering of host use relative to null expectations.


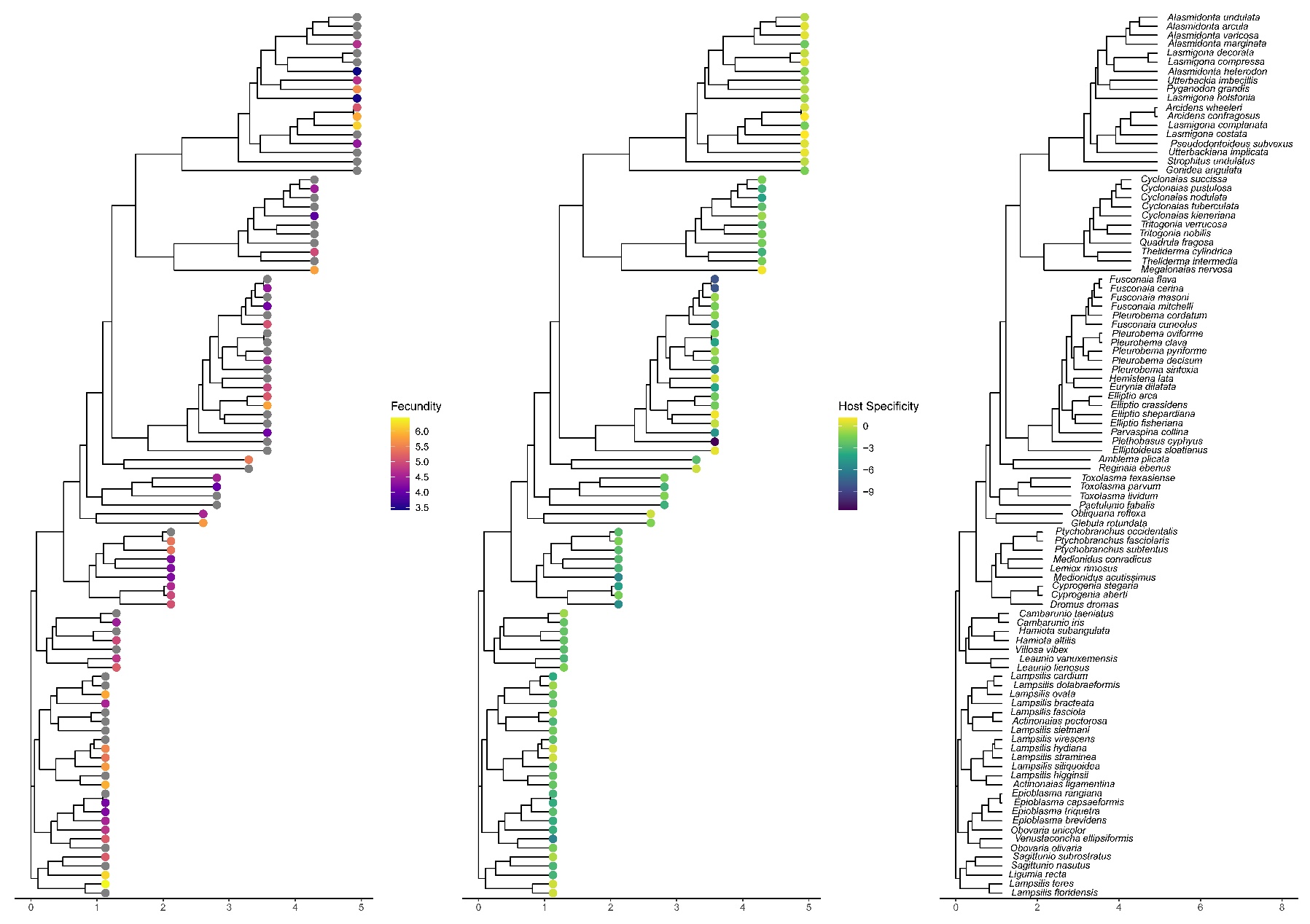


Figure S2. Phylogenetic tree of freshwater mussel species with trait data visualized at the tips: left shows fecundity, center shows host specificity (log-transformed), and right displays species names for reference.


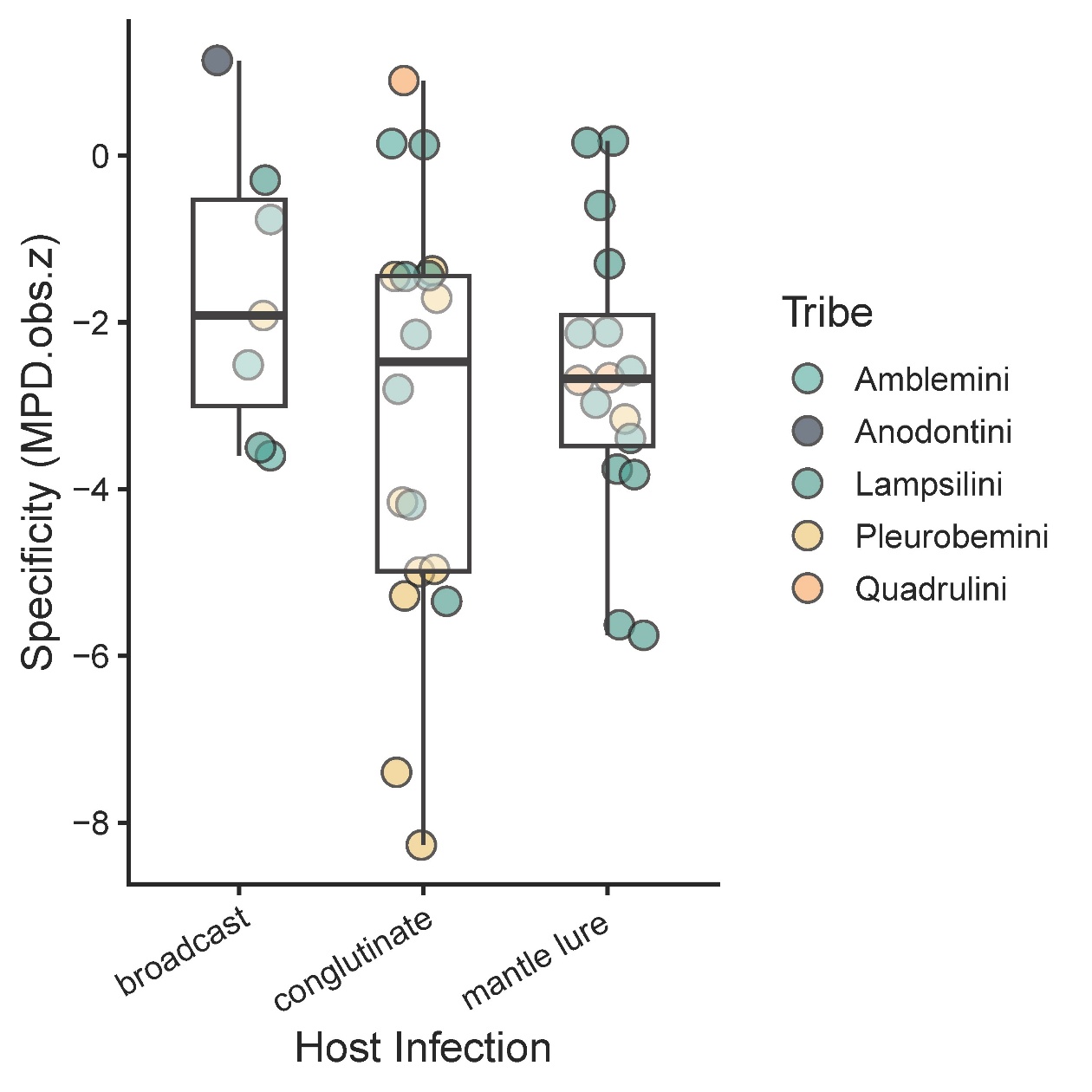


Figure S3. Specificity (MPD. Obs Z) of freshwater mussel lineages across host infection strategies. Boxplots summarize the distribution of specificity for broadcast, conglutinate, and mantle lure infections, with points representing individual species. Colors indicate mussel tribe (Amblemini, Anodontini, Lampsilini, Pleurobemini, Quadrulini). Lower specificity values indicate greater phylogenetic clustering of host use.


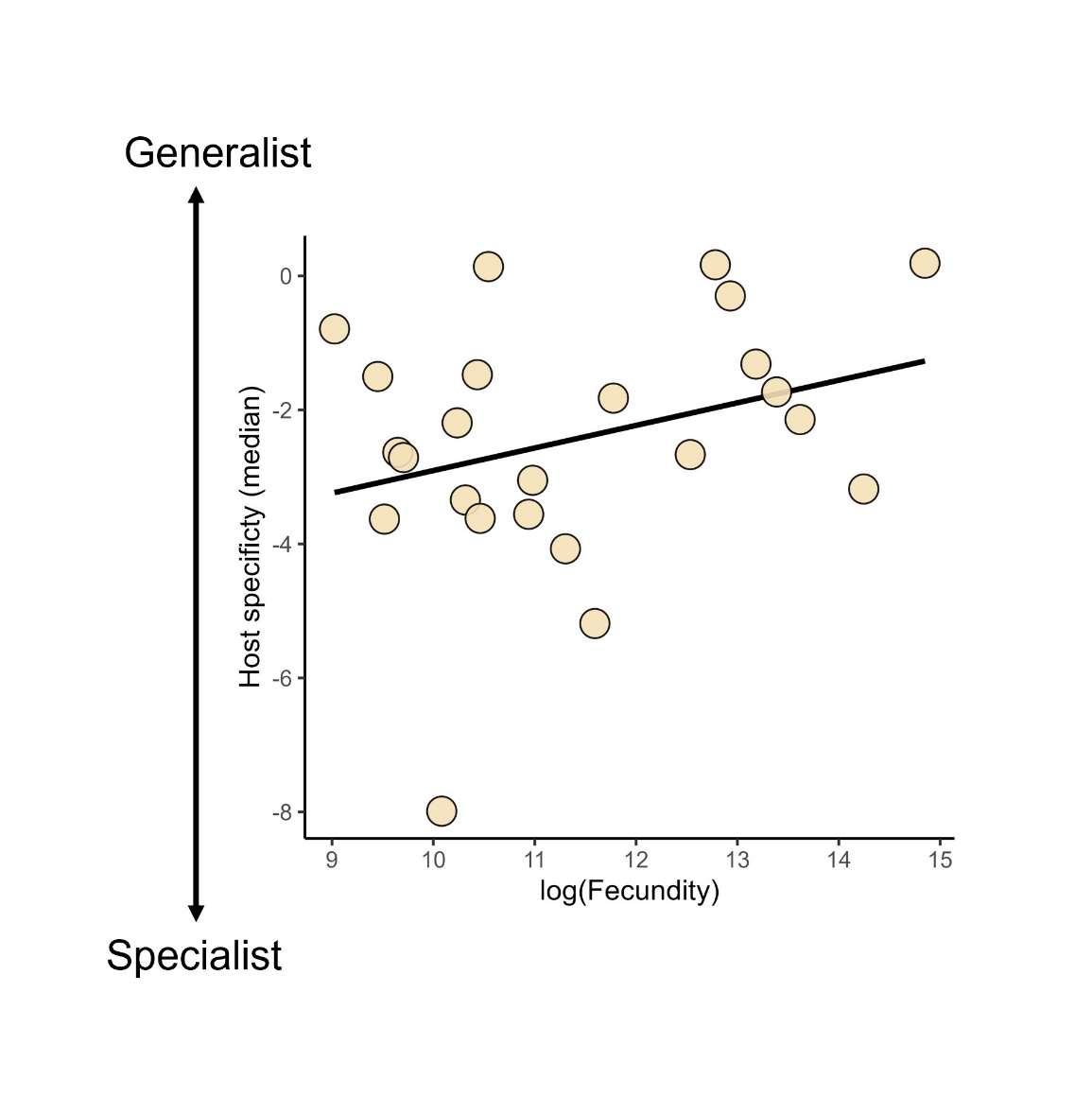


Figure S4. Scatterplot showing the relationship between fecundity (log-transformed for visual clarity) and host phylogenetic specificity using a Phylogenetic Generalized Least Square model. Each point represents a mussel species included in the model.
